# Reply: Metabolic Heterogeneity of Obesity and Outcome-Specific Effects of GLP-1 Receptor Agonists in Cardiovascular-Kidney-Metabolic Syndrome

**DOI:** 10.1016/j.jacadv.2026.102806

**Published:** 2026-05-14

**Authors:** Sonal Kumar, John E. Anderson, Andrea Coviello, Francisco Lopez-Jimenez

**Affiliations:** a Weill Cornell Medical College, Cornell University, New York, New York, USA; b The Frist Clinic, Nashville, Tennessee, USA; c University of North Carolina, Chapel Hill, North Carolina, USA; d Mayo Clinic, Rochester, Minnesota, USA

We thank Drs Fauchier and Morel for their thoughtful response to our review of the role of glucagon-like peptide 1 receptor agonists (GLP-1 RAs) in cardiovascular-kidney-metabolic syndrome,^1^ and for highlighting the importance of personalizing GLP-1 RA use. We agree that identifying those patients most likely to derive benefit from this drug class is of significant interest to the medical community.

The correspondents raise the distinction in cardiovascular risk between individuals classed as having metabolically healthy obesity (MHO) vs metabolically unhealthy obesity (MUHO). Although this is an important consideration, several limitations of the MHO/MUHO concept should be acknowledged.^2^ Firstly, despite growing research interest, there is no universally accepted definition of MHO, with heterogeneity in criteria and thresholds across studies that investigate this condition.^2^ Secondly, MHO is increasingly recognized as a dynamic rather than stable phenotype, with up to 50% of people with MHO transitioning to the metabolically unhealthy phenotype, particularly with increasing age.^2^ Finally, even MHO is associated with an elevated risk of cardiovascular disease, type 2 diabetes, and all-cause mortality compared with lean individuals, suggesting that MHO should not be considered a benign condition.^2^

An important consideration when evaluating GLP-1 RA use in those with any form of obesity is that benefits may extend beyond cardiometabolic events to improvements in mechanical complications, such as obstructive sleep apnea and osteoarthritis.^3^

Dr Fauchier and Dr Morel are correct to highlight emerging evidence suggesting that the reduction in cardiovascular events associated with GLP-1 RA use may be greater in individuals with MUHO compared with MHO.^4^ Our review article primarily referred to whole population outcomes from key phase 2 and 3 clinical trials of GLP-1 RAs.^1^ Relatively few subgroup analyses stratified by baseline metabolic characteristics are currently available, and meta-analyses incorporating such factors are also, to our knowledge, scarce. However, there are some exceptions. For example, in a prespecified analysis of the SELECT trial, which investigated the role of once-weekly semaglutide in people with overweight or obesity in reducing cardiovascular events, lower baseline waist circumference—an indicator of visceral adiposity—and lower baseline bodyweight were associated with lower risk of major adverse cardiovascular events.^5^ Indeed, waist circumference reduction was calculated as mediating only ∼33% of the risk reduction associated with semaglutide treatment, suggesting that the cardiovascular benefits of GLP-1 RAs may be driven largely by mechanisms beyond weight loss alone.^5^

Overall, GLP-1 RAs appear to confer broad clinical benefits in individuals with obesity. Nevertheless, a more nuanced understanding of metabolic health in the context of GLP-1 RAs will be essential to optimizing their use in clinical practice. Although existing subgroup analyses offer preliminary insights into populations that may derive greater benefit, these analyses are often underpowered and exploratory in nature. Addressing these gaps will require a combination of new clinical trials, subgroup analyses, and high-quality real-world evidence.
